# Long Non-Coding RNA-Mediated Competing Endogenous RNA Networks in Ischemic Stroke: Molecular Mechanisms, Therapeutic Implications, and Challenges

**DOI:** 10.3389/fphar.2021.765075

**Published:** 2021-11-17

**Authors:** Shuxia Zhang, Ting Zhu, Qiaoyu Li, Guibo Sun, Xiaobo Sun

**Affiliations:** ^1^ Beijing Key Laboratory of Innovative Drug Discovery of Traditional Chinese Medicine (Natural Medicine) and Translational Medicine, Institute of Medicinal Plant Development, Peking Union Medical College and Chinese Academy of Medical Sciences, Beijing, China; ^2^ Key Laboratory of Bioactive Substances and Resources Utilization of Chinese Herbal Medicine, Ministry of Education, Institute of Medicinal Plant Development, Chinese Academy of Medical Sciences and Peking Union Medical College, Beijing, China; ^3^ Key Laboratory of Efficacy Evaluation of Chinese Medicine Against Glycolipid Metabolic Disorders, State Administration of Traditional Chinese Medicine, Institute of Medicinal Plant Development, Peking Union Medical College and Chinese Academy of Medical Sciences, Beijing, China; ^4^ Zhongguancun Open Laboratory of the Research and Development of Natural Medicine and Health Products, Institute of Medicinal Plant Development, Chinese Academy of Medical Sciences and Peking Union Medical College, Beijing, China; ^5^ Key Laboratory of New Drug Discovery Based on Classic Chinese Medicine Prescription, Chinese Academy of Medical Sciences, Beijing, China; ^6^ Institute of Neuroregeneration and Neurorehabilitation, Qingdao University, Qingdao, China

**Keywords:** long non-coding RNA, competing endogenous RNA network, ischemic stroke, ischemia-reperfusion injury, therapeutic target

## Abstract

Ischemic stroke (IS) is a disease that is characterized by high mortality and disability. Recent studies have shown that LncRNA-mediated competing endogenous RNA (ceRNA) networks play roles in the occurrence and development of cerebral I/R injury by regulating different signaling pathways. However, no systematic analysis of ceRNA mechanisms in IS has been reported. In this review, we discuss molecular mechanisms of LncRNA-mediated ceRNA networks under I/R injury. The expression levels of LncRNAs, microRNAs (miRNAs), and messenger RNAs (mRNAs) and their effects in four major cell types of the neurovascular unit (NVU) are also involved. We further summarize studies of LncRNAs as biomarkers and therapeutic targets. Finally, we analyze the advantages and limitations of using LncRNAs as therapeutics for IS.

## Introduction

Ischemic stroke (IS) is a neurological disorder that is characterized by blockage of blood vessels and accounts for approximately 87% of strokes (Fann et al., 2013; Lin et al., 2016; Kuriakose and Xiao 2020). Currently, the accepted therapeutic strategy for IS is vascular recanalization therapy which including thrombolysis (with agents such as tissue plasminogen activators), mechanical thrombectomy, and the combination of them. However, reperfusion therapy must be applied within a very short period, which drastically limits the population that is eligible for treatment. Moreover, ischemia-reperfusion (I/R) injury occurs when blood is resupplied to cerebral ischemic tissues, as it is harmful to blood vessels and brain cells such as brain microvascular endothelial cells (BMECs), neurons, and microglial cells. The extent of the ensuing lesion is dependent on the active and complicated interaction between vascular cells, neurons, and glial cells (Trendelenburg and Dirnagl 2005). The precise mechanisms underlying I/R injury remain unknown. However, oxidative stress, inflammation, autophagy, apoptosis, and blood-brain barrier (BBB) disruption are potential mechanisms (Zhu et al., 2020).

Long non-coding RNAs (LncRNAs), a class of non-coding RNAs that are more than 200 nucleotides in length (Kapranov et al., 2007), have been widely studied. It has been reported that LncRNAs affect the occurrence and outcome of several diseases by regulating chromatin modification, post-transcription, and transcription (Ma et al., 2013; McDonel and Guttman 2019; Nair et al., 2020). LncRNAs are also involved in the pathological progression of tumors, nervous system disorders, cardiovascular diseases, and other diseases (Schmitz et al., 2016; Peng et al., 2017; Huang 2018; Wolska et al., 2020). Recent studies have shown that LncRNAs, such as lncRNA metastasis-associated lung adenocarcinoma transcript 1 (MALAT1), play important roles in IS (Zhang et al., 2017a). Another class of non-coding RNAs is microRNAs (miRNAs), which are single-stranded endogenous RNAs with a length of 19–25 nt (Lu and Rothenberg 2018). It has been shown that miRNAs function by targeting the 3ʹ-untranslated (3ʹ-UTR) region of messenger RNAs (mRNAs), thus inhibiting protein synthesis or promoting mRNA degradation (Kabekkodu et al., 2018). Since miRNAs are involved in disease development, they have been a potential target for therapeutic approaches, especially in cancer management (Rupaimoole and Slack 2017; Fan et al., 2019).

**FIGURE 1:**
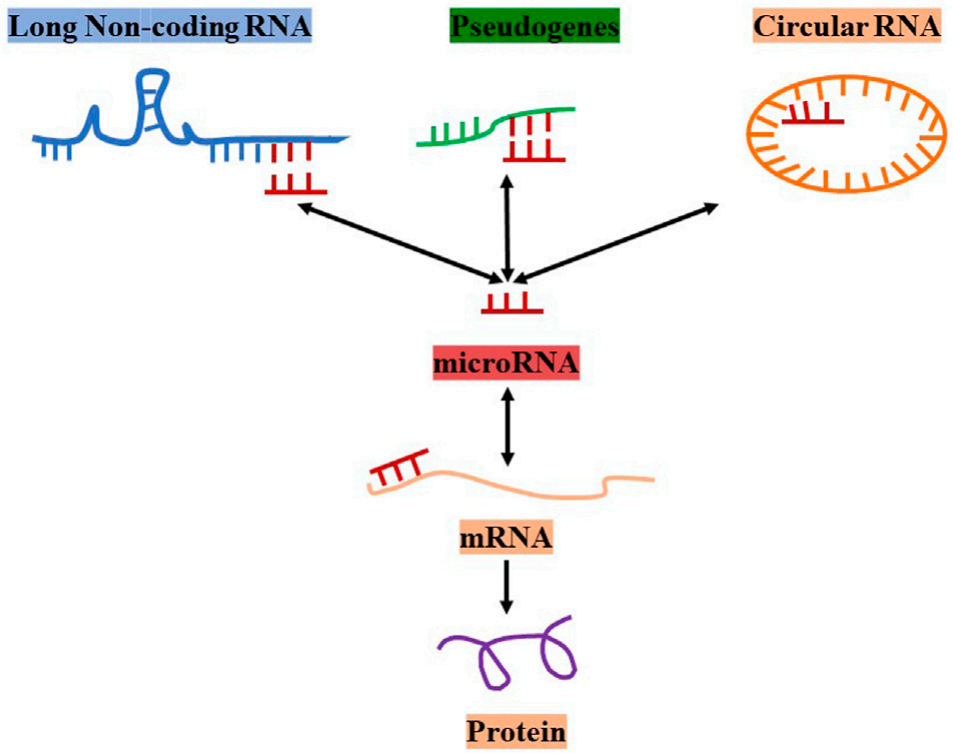
The concept and types of ceRNAs.

The competing endogenous RNA (ceRNA) hypothesis was first proposed by Salmena et al. in 2011 (Salmena et al., 2011). The concept suggests that there are ceRNAs such as LncRNAs, circular RNAs, pseudogenes, and mRNAs in cells. Additionally, ceRNAs can competitively bind to the same miRNA by interacting with miRNA response elements (MREs) to build communication networks between messenger RNAs and non-coding RNAs (Salmena et al., 2011; Sanchez-Mejias and Tay 2015). Thus, LncRNAs can compete with an mRNA, bind to the same miRNA, and regulate the expression of the mRNA if they have the same MRE (Figure 1).

In this review, we focus on LncRNAs that mediate ceRNA networks and regulate key processes involved in I/R injury to explore the potential roles of LncRNAs in regulating IS. We have also summarized the molecular mechanisms of the LncRNA-miRNA-mRNA axis in autophagy, apoptosis, angiogenesis, microglial activation, and polarization in different cell lines (Table 1). Furthermore, we have discussed the therapeutic implications and challenges of LncRNAs in IS.

**TABLE 1:** Studies evaluating LncRNA-mediated ceRNA networks in IS.

Reference	LncRNA	Model	Pathophysiological mechanism	Axis	Effects
Yan et al. (2017)	LncRNA MEG3	C57BL/6 J mice, N2a cells	Apoptosis	LncRNA MEG3/miR-21/PDCD4	MEG3 aggravated apoptosis of neurons and ischemic damage
Liang et al. (2020)	LncRNA MEG3	Sprague Dawley (SD) rat, SK-N-SH, SH-SY5Y cells	Pyroptosis, inflammation	LncRNA MEG3/miR-485/AIM2/caspase-1	MEG3 contributed to pyroptosis and inflammation *via* miR-485/AIM2 in cerebral I/R injury
Luo et al. (2020)	LncRNA MEG3	C57BL/6J mice, primary mouse cortical neurons	Autophagy	LncRNA MEG3/miR-378/GRB2/Akt/mechanistic target of rapamycin (mTOR)	Knockdown of LncRNA MEG3 resulted in reduced autophagy *via* targeting of the miR-378/GRB2/Akt/mTOR axis
Xiang et al. (2020)	LncRNA MEG3	C57BL/6J mice, SD rats, N2a cells	Apoptosis	LncRNA MEG3/miR-424-5p/Sema3A/MAPK pathway	Downregulation of LncRNA MEG3 expression resulted in reduced apoptosis and suppressed MAPK pathway *via* targeting of miR-424-5p and then modulation of Sema3A expression
Xu et al. (2021)	LncRNA H19	C57BL/6J mice, HT22 cells	Apoptosis, inflammation	LncRNA H19/miR-29b/SIRT1/PGC-1α	Knockdown of LncRNA H19 resulted in ameliorated OGD-induced inflammation and apoptosis *via* regulation of the miR-29b/SIRT1/PGC-1α axis
Xiao et al. (2019)	LncRNA H19	Patients with anterior circulation ischemia, SH-SY5Y cells, human embryonic kidney (HEK) 293T cells, SD rats	Apoptosis	LncRNA H19/miR-19a/Id2	Suppression of LncRNA H19 expression resulted in protection against neuronal injury induced by hypoxia/ischemia *via* regulation of the miR-19a/Id2 axis
Chen et al. (2018)	LncRNA GAS5	C57BL/6J mice, mouse primary brain neurons	Apoptosis	LncRNA GAS5/miR-137/Notch1 signaling pathway	GAS5 functioned as a ceRNA for miR-137 to regulate Notch1 and promote neuronal injury by inhibiting the Notch1 signaling pathway
Wu et al. (2021)	LncRNA GAS5	SD rats, PC12 cells	Apoptosis, mitochondrial damage	LncGAS5/miR-455-5p/PTEN	Upregulation of GAS5 expression resulted in downregulated miR-455-5p expression, which promoted PTEN expression, aggravated mitochondrial damage, worsened neurobehavior, and promoted apoptosis and oxidative injury
Zhou et al., (2020)	LncRNA SNHG7	C57BL/6J mice, PC12 cells	Apoptosis	LncRNA SNHG7/miR-9/SIRT1	LncRNA SNHG7 served as a ceRNA for miR-9 to regulate SIRT1 expression, thus alleviating neuronal injury
Yan et al. (2020b)	LncRNA SNHG12	SH-SY5Y cells	Apoptosis	SNHG12/miR-181a-5p/NEGR1	SNHG12 inhibited OGD-induced apoptosis of SH-SY5Y cells *via* the miR-181a-5p/NEGR1 axis
Guo et al. (2020)	LncRNA SNHG15	C57BL/6J mice, N2a cells	Apoptosis	LncRNA SNHG15/miR-18a/CXCL13/ERK/MEK	Silencing of SNHG15 resulted in enhanced viability and reduced apoptosis of N2a cells *via* the miR-18a/CXCL13/ERK/MEK axis
Fan et al. (2021)	LncRNA SNHG15	PC12 cells	Apoptosis, inflammation	LncRNA SNHG15/miR-455-3p/TP53INP1	Knockdown of SNHG15 resulted in protection against OGD/R-induced neuronal injury *via* regulation of the miR-455-3p/TP53INP1 axis
Zhao et al. (2021)	LncRNA RMST	IS patients, N2a cells	Apoptosis	RMST/miR-377/SEMA3A	Upregulating RMST expression promoted apoptosis and oxidative stress *via* regulation of the miR-377/SEMA3A axis in IS patients
Cheng et al. (2020)	LncRNA RMST	HT-22 cells	Apoptosis	RMST/hnRNPK/p53/miR-107/Bcl2l2	The RMST/hnRNPK/p53/miR-107 axis inhibited Bcl2l2 expression and promoted apoptosis
Guo et al. (2017)	LncRNA MALAT1	C57BL/6J mice, cerebral cortex neurons	Autophagy	LncRNA MALAT1/MiR-30a/Beclin-1	Suppressing MALAT1 expression attenuated neuronal death *via* sponging of miR-30a to regulate Beclin-1 expression in cerebral IS
Jia et al. (2021)	LncRNA MALAT1	HT-22 cells	Apoptosis	LncRNA MALAT1/miR-195a-5p/HMGA1	MALAT1 promoted neuronal injury through binding to miR-195a-5p and then by upregulating HMGA1 expression
Zhang et al. (2020a)	LncRNA MALAT1	SD rats, PC12 cells	Inflammation, apoptosis	LncRNA MALAT1/miR-375/PDE4D	Suppressing MALAT1 expression alleviated inflammation and apoptosis in a rat model of MCAO/R through modulation of the miR-375/PDE4D axis
Liu et al. (2019)	LncRNA ANRIL	PC12 cells	Apoptosis	LncRNA ANRIL/miR-127/Mcl-1	LncRNA ANRIL alleviated OGD-induced PC12 cell injury
Zhong et al. (2020)	LncRNA ANRIL	C57BL/6J mice, N2a cells	Apoptosis	LncRNA ANRIL/miR-199a-5p/CAV-1/phosphorylation of MEK/ERK	ANRIL protected N2a cells by sponging miR-199a-5p, thus downregulating CAV-1 expression and activating the MEK/ERK pathway
Chen et al. (2017)	LncRNA TUG1	SD rats, Primary cortical neurons, SH-SY5Y cells	Apoptosis	LncRNA TUG1/miRNA-9/Bcl2l11	TUG1 sponges miR-9 to aggravate neuronal apoptosis by upregulating Bcl2l11 expression
Wang et al. (2018a)	LncRNA HOTTIP	C57BL/6J mice, Primary cortical neurons from mouse embryos	Apoptosis, glycolytic metabolism	LncRNA HOTTIP/miR-143/hexokinase 2	HOTTIP reduced OGD/R-induced neuronal injury by regulating the miR-143/hexokinase 2 axis
Cai et al. (2019)	LncRNA Gm11974	HEK293T cells, N2a cells	Apoptosis	LncRNA Gm11974/miR-766-3p/NR3C2	Knockdown of Gm11974 resulted in protection against neuronal apoptosis *via* the miR-766-3p/NR3C2 axis
Gai et al. (2019)	LncRNA CHRF	C57BL/6J mice, N2a cells	Apoptosis	CHRF/miR-126/SOX6	Knockdown of CHRF resulted in reduced ischemic neuronal death *via* upregulation of miR-126 expression and reduction in SOX6 expression
Jing et al. (2019)	LncRNA Oprm1	C57BL/6J mice, N2a cells	Apoptosis	Oprm1/miR-155/GATA3 axis	Oprm1 played a protective role in cerebral stroke by acting as a ceRNA for miR-155 to target GATA3
Wei et al. (2019)	LncRNA AK038897	C57BL/6J mice, N2a cells	Apoptosis	AK038897/miR-26a-5p/DAPK1	LncRNA AK038897 aggravated cerebral I/R injury by regulating the expression of miR-26a-5p and DAPK1
Zhang et al. (2019b)	LncRNA FGD5-AS1	Primary cortical neurons isolated from the brains of SD rats	Apoptosis	FGD5-AS1/miRNA-223/IGF1R	FGD5-AS1 acted as a protective factor in OGD/R-induced neuronal injury *via* competitive binding to miR-223 to regulate IGF1R expression
Lu et al. (2020)	LncRNA FOXD3-AS1	C57BL/6J mice, N2a cells	Apoptosis	FOXD3-AS1/miR-765/BCL2L13	Knockdown of FOXD3-AS1 resulted in protection against cerebral I/R injury *via* binding to miR-765 to regulate BCL2L13 expression
Yao et al. (2020)	LncRNA Rian	C57BL/6J mice, N2a cells	Apoptosis	Rian/miR-144-3p/GATA3	Overexpression of LncRNA Rian caused a reduction in apoptosis induced by cerebral I/R injury *via* the miR-144-3p/GATA3 axis
Zhang et al. (2019a)	LncRNA SNHG6	C57BL/6J Mice, primary mouse cortical neurons	Apoptosis	LncRNA SNHG6/miR-181c-5p/BIM	SNHG6 functioned as a ceRNA to regulate neuronal apoptosis by regulating the miR-181c-5p/BIM axis in IS
Cao et al. (2020a)	LncRNA TALNEC2	C57BL/6J mice, N2a cells	Apoptosis	TALNEC2/miR-650/APAF1	TALNEC2 aggravated cerebral I/R injury by serving as a ceRNA for miR-650 to target APAF1
Zhou et al. (2020)	LncRNA GAS5	SD rats, HEK293 cells, primary rat cortical cells, B35 cell line	Apoptosis	GAS5/miR-221/p53 upregulated modulator of apoptosis (PUMA) axis	GAS5 aggravated apoptosis induced by OGD/R injury by regulating the miR-221/PUMA axis
Yu et al. (2019)	KCNQ1OT1	Acute IS patients, C57BL/6J mice, N2a cells	Autophagy	KCNQ1OT1/miR-200a/FOXO3/ATG7 pathway	KCNQ1OT1 promoted autophagy by modulating the miR-200a/FOXO3/ATG7 axis in IS
Yi et al. (2020)	KCNQ1OT1	IS Patients, PC12 cells	Apoptosis	KCNQ1OT1/miR-140-3p/HIF-1α	KCNQ1OT1 exacerbates apoptosis and I/R injury by regulating the miR-140-3p/HIF-1α axis
Li et al. (2017b)	LncRNA MALAT1	BMECs, C57BL/6J mice	Autophagy	LncRNA MALAT1/miR-26b/ULK2	MALAT1 promoted BMEC autophagy and survival by working as a ceRNA to sponge miR-26b and by upregulating ULK2 expression
Teng et al. (2020)	LncRNA SNHG16	HBMECs	Apoptosis	LncRNA SNHG16/miR-15a-5p/Bcl2	SNHG16 protected against OGD/R-induced apoptosis of HBMECs by regulating the miR-15a-5p/Bcl2 axis
Yin et al. (2021)	LncRNA RMST	HBMECs, bEnd.3 cells	Apoptosis	RMST/miR-204-5p/VCAM1	Knockdown of RMST resulted in reduced OGD-induced injury *via* regulation of the miR-204-5p/VCAM1 axis
Chen et al. (2020)	LncOGD-1006	Primary BMECs	Apoptosis	LncOGD-1006/miR-184-5p/CAAP1	LncOGD-1006 alleviates OGD-induced apoptosis *via* the miR-184-5p/CAAP1 axis
Li et al. (2017a)	LncRNA HIF1A-AS2	SD rats, HUVECs	Angiogenesis	LncRNA HIF1A-AS2/miR-153-3p/HIF-1α/VEGFA/Notch1	HIF1A-AS2 promoted angiogenesis in HUVECs by binding to miR-153-3p to upregulate HIF-1α expression
Zhao et al. (2018)	LncRNA SNHG12	C57BL/6J mice, bEnd.3 cells	Angiogenesis	SNHG12/miR-150/VEGF	SNHG12 played a protective role in angiogenesis reduction in IS by regulating the miR-150/VEGF axis
Wang et al. (2018b)	LncRNA SNHG1	C57BL/6J mice, BMECs	Angiogenesis	LncRNA SNHG1/miR-18a/HIF-1α/VEGF	SNHG1 played a protective role in angiogenesis as a ceRNA through HIF-1α/VEGF signaling in IS
Yang et al. (2018)	LncRNA SNHG1	BMECs	Angiogenesis	LncRNA SNHG1/miR-338/HIF-1α/VEGF	SNHG1 protected BMECs against OGD-induced injury by regulating the miR-338/HIF-1α/VEGF-A axis
Yan et al. (2020a)	LncRNA MACC1-AS1	HBMECs	Angiogenesis	LncRNA MACC1-AS1/miR-6867-5p/TWIST1	MACC1-AS1 protected hypoxic HBMECs by promoting angiogenesis *via* regulation of the miR-6867-5p/TWIST1 axis
Zhang et al. (2020b)	LncRNA DANCR	BMECs	Angiogenesis	LncRNA DANCR/miR-33a-5p/XBP1s	DANCR promoted cell survival and angiogenesis by regulating the miR-33a-5p/XBP1s axis
Gao and Wang (2020)	LncRNA MALAT1	bEnd.3 cells, HEK293T cells	Apoptosis	LncRNA MALAT1/miR-205-3p/PTEN	MALAT1 suppressed apoptosis in IS and functioned as a ceRNA for miR-205-3p to modulate PTEN expression
Gao et al. (2020a)	LncRNA MALAT1	HBMECs	Angiogenesis	LncRNA MALAT1/miR-205-5p/VEGFA	MALAT1 promoted angiogenesis in HBMECs subjected to OGD/R by interacting with the miR-205-5p/VEGFA axis
Tian et al. (2021)	LncRNA Snhg8	BMECs, primary microglial cells	Microglial inflammation, BBB disruption	LncRNA Snhg8/miR-425-5p/SIRT1/NF-κB pathway	Snhg8 inhibited microglial activation and alleviated BMEC injury by sponging miR-425-5p and regulating the SIRT1/NF-κB axis
Zhang et al. (2020a)	LncRNA SNHG14	C57BL/6J mice, BV2 cells	Inflammation	LncRNA SNHG14/miR-199b/AQP4 Axis	SNHG14 knockdown resulted in reduced inflammation and oxidative stress *via* regulation of the miR-199b/AQP4 axis
Qi et al. (2017)	LncRNA SNHG14	C57BL/6J mice, microglia, neurons	Microglial activation, apoptosis	LncRNA SNHG14/miR-145-5p/PLA2G4A	SNHG14 promoted microglial activation and apoptosis by regulating the miR-145-5p/PLA2G4A pathway
Chen et al. (2021b)	LncRNA OIP5-AS1	SD rats, microglial cells	Inflammation, neuronal apoptosis, oxidative stress	OIP5-AS1/miR-186-5p/CTRP3 axis	Upregulation of OIP5-AS1 expression resulted in protection against neuronal injury in MCAO/R-induced inflammation and oxidative stress in microglia/macrophages *via* CTRP3 activation and interaction with miR-186-5p
Wang et al. (2020a)	LncRNA MALAT1	C57BL/6J mice, astrocytes	Apoptosis	LncRNA MALAT1/miR-145/AQP4	LncRNA MALAT1 aggravated apoptosis by improving AQP4 expression *via* miR-145 sponging
Song et al. (2021)	KCNQ1OT1	HMC3 cells	Inflammation, apoptosis	KCNQ1OT1/miR-30e-3p/NLRP3	Inhibition of KCNQ1OT1 reduced the inflammation and apoptosis *via* miR-30e-3p/NLRP3 pathway
Ren et al. (2020)	KCNQ1OT1	Primary mouse cerebral cortical neurons	Apoptosis	KCNQ1OT1/miR-9/MMP8 axis	Inhibition of KCNQ1OT1 possibly ameliorated neuronal injury by regulating miR-9/MMP8 axis
Wang et al. (2020b)	KCNQ1OT1	Primary mouse cerebral cortical neurons	Apoptosis	KCNQ1OT1/miR-153-3p/FOXO3 Axis	KCNQ1OT1 promoted neuronal injury through regulating Foxo3 expressions *via* miR-153-3p
Zhong et al., (2019)	LncRNA SNHG14	C57BL/6J mice	Inflammation	LncRNA SNHG14/miR-136-5p/ROCK1	SNHG14 promoted neurological impairment and inflammatory response *via* miR-136-5p/ROCK1 axis
		SD rats, PC-12 cells			
Deng et al. (2020)	LncRNA SNHG14	HT22 cells	Mitophagy	LncRNA SNHG14/miR-182-5p/BINP3	LncRNA SNHG14 promoted neuron injury by regulating mitophagy *via* miR-182-5p/BINP3 axis
Li et al. (2020)	LncRNA H19	SD rats, PC-12 cells	Inflammation	LncRNA H19/miR-138-5p/p65	LncRNA H19 promoted inflammation by regulating miR-138-5p/p65 axis
Gao et al. (2020a)	LncRNA H19	C57BL/6J mice, SH-SY5Y cells	Oxidative stress, apoptosis	LncRNA H19/miR-19a-3p/PTEN axis	LncRNA H19 promoted Oxidative stress and apoptosis induced by I/R or OGD/R through miR-19a-3p/PTEN axis
Shan et al., (2020)	LncRNA TUG1	C57BL/6J mice, MA-C cells	Apoptosis	LncRNA TUG1/miR-145/AQP4	LncRNA TUG1 aggravated apoptosis by improving AQP4 expression *via* miR-145 sponging
Cao et al. (2020b)	LncRNA Malat1	C57BL/6J mice、BV-2 cells	Inflammation	LncRNA Malat1/miR-181c-5p/HMGB1	LncRNA Malat1/miR-181c-5p/HMGB1 axis may play a vital role in poststroke inflammation
Cao et al. (2020b)	LncRNA Malat1	C57BL/6J mice、BV-2 cells	Inflammation	LncRNA Malat1/miR-181c-5p/HMGB1	LncRNA Malat1/miR-181c-5p/HMGB1 axis may play a vital role in poststroke inflammation

In the axis column, upregulated actors are in red font, downregulated factors are in blue font, whereas unknown factors are in black font.

## Article Search Process

**FIGURE 2:**
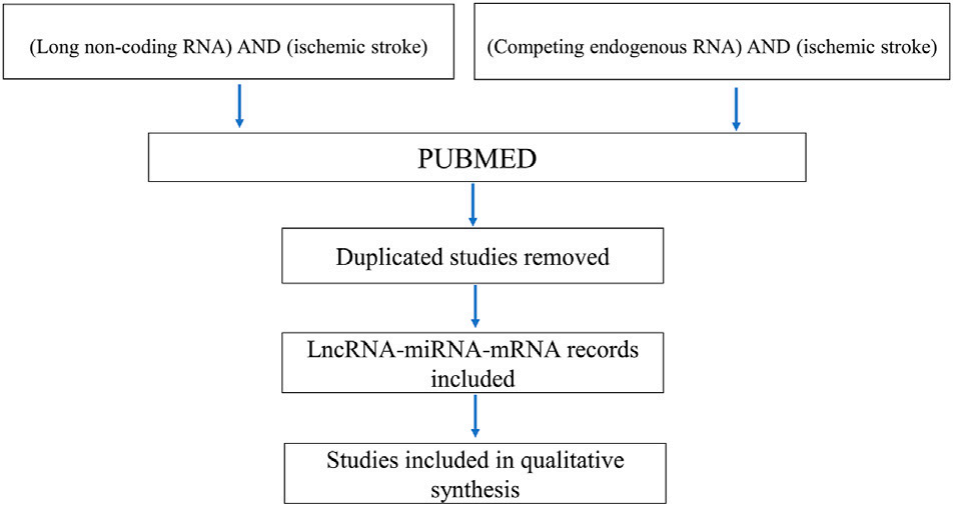
Article selection process.

Studies included in the review were obtained by searching the PubMed database. The following search syntaxes were used (long non-coding RNA) AND (ischemic stroke) and (competing endogenous RNA) AND (ischemic stroke). Review articles and meta-analyses were included for possible support (Figure 2).

## Regulatory Roles of LncRNAs in Neurons

**FIGURE 3:**
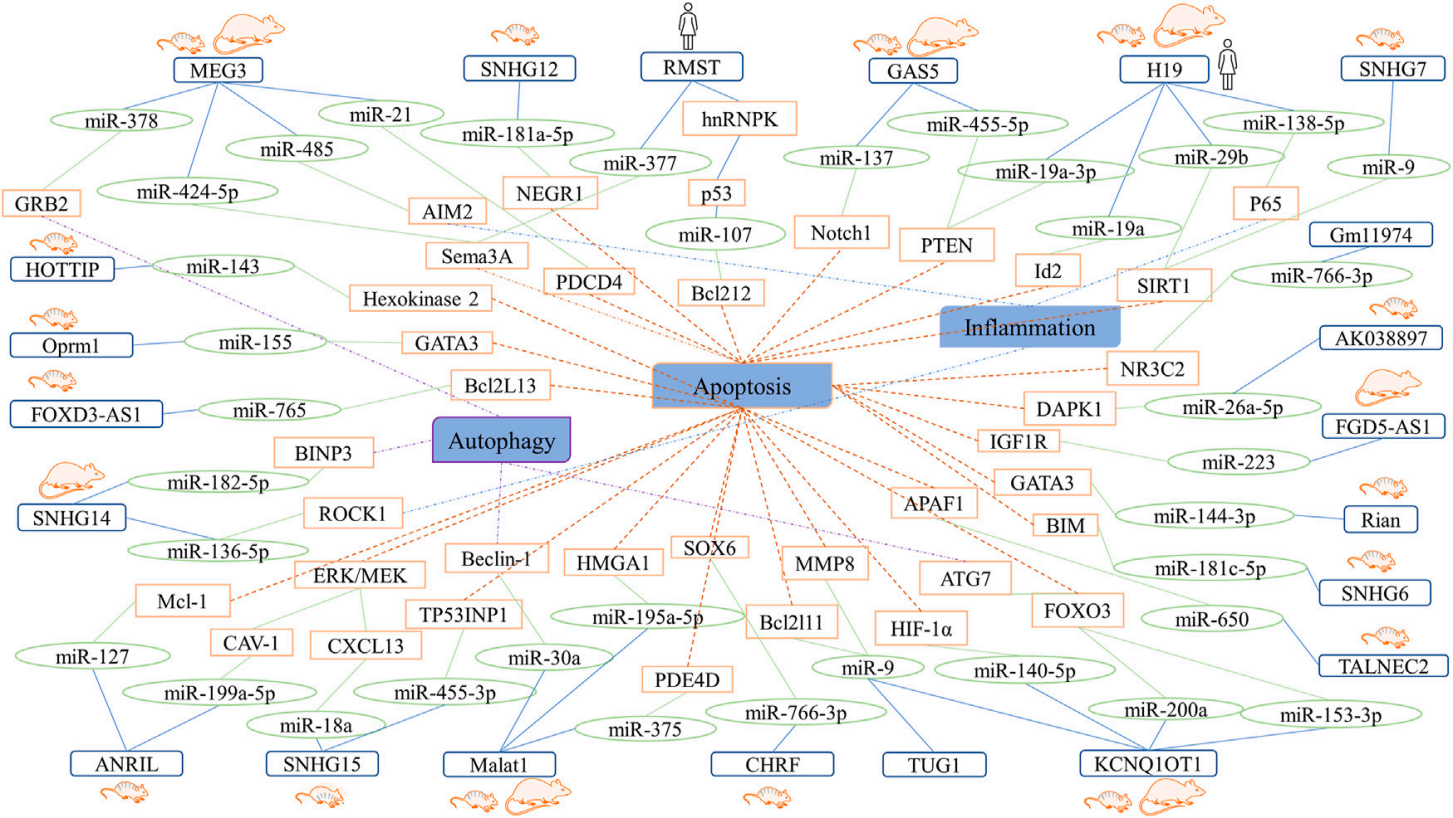
Summary network showing the roles of LncRNA-miRNA-mRNA axes in neurons.

The focal areas of IS are the ischemic core area and the penumbra. In the ischemic core area, failure of the ion pump of the neuronal cell membrane and energy metabolism leads to irreversible damage to the brain tissue. However, there are collateral circulations and surviving neurons in the ischemic penumbra. Thus, restoring blood supply to the ischemic penumbra as soon as possible and using effective neuroprotective drugs are important research aspects in IS. Furthermore, prevention and inhibition of neuronal cell injury are potential treatment strategies. In this section, we summarize the effects and ceRNA networks of LncRNAs in neurons and provide evidence for the development of RNA drugs (Figure 3).

### poptosis

#### LncRNA Maternally Expressed Gene 3

In a previous study, LncRNA MEG3 expression was upregulated in middle cerebral artery occlusion/reperfusion (MCAO/R) models, which mainly protected ischemic neurons. Additionally, MEG3 served as a ceRNA for microRNA-21 (miR-21), which was downregulated *in vivo* and *in vitro* in contrast to MEG3. Furthermore, programmed cell death 4 (PDCD4), a gene that mediates neuronal cell death, binds to miR-21. Overexpression of miR-21 resulted in protection against oxygen-glucose deprivation and reoxygenation (OGD/R)-induced apoptosis, whereas knockdown of MEG3 resulted in protection against I/R injury and improved neurological function in IS patients (Yan et al., 2017). Similarly, Liang et al. observed that inhibition of MEG3 expression contributed to pyroptosis *via* activation of the miR-485/absent in melanoma 2 (AIM2)/caspase-1 pathway (Liang et al., 2020). Moreover, MEG3 targets miR-424-5p *via* neuronal apoptosis mediated *via* the mitogen-activated protein kinase (MAPK) pathway (Xiang et al., 2020).

#### LncRNA H19

In a previous study, LncRNA H19 expression was found to be upregulated in SD rats, C57 mice, and an OGD cell culture model. Additionally, H19 siRNA improved apoptosis and inflammation and influenced the expression of miR-29b, sirtuin1 (SIRT1), and peroxisome proliferator-activated receptor-gamma coactivator (PGC)-1α expression in OGD cell culture models (Xu et al., 2021). Another report indicated that H19 levels were elevated in patients with anterior circulation ischemia and that H19 knockdown resulted in reduced apoptosis in OGD neuronal cells. Furthermore, inhibition of H19 expression in a rat model of MCAO/R resulted in a marked decrease in brain infarct volume, neurological deficits, and neuronal apoptosis. It was also found that H19 plays a critical role in neuronal apoptosis as a ceRNA by interfering with the binding of inhibitor of DNA binding 2 (Id2) and PTEN to miR-19a and miR-19a-3p (Xiao et al., 2019; Gao N. et al., 2020).

#### LncRNA Growth Arrest-specific 5

LncRNA GAS5 expression is attributed to negative regulation of cell survival. Upregulation of GAS5 expression has been demonstrated both *in vivo* and *in vitro*. It has also been reported that GAS5 knockdown results in a significant increase in cell viability, suppression of caspase-3 activation, and the induction of neuronal apoptosis after OGD. Moreover, GAS5 serves as a molecular sponge for miR-137 to regulate Notch1 expression and reduce neuron survival (Chen et al., 2018). Wu et al. revealed the functions of the GAS5/miR-455-5p/phosphatase and tension homolog deleted on chromosome ten (PTEN) axis in cerebral IS. It was found that GAS5 and PTEN levels were upregulated whereas miR-445-5p level was downregulated in brain and PC12 cell models of MCAO/R injury that were subjected to OGD/R. Additionally, suppression of GAS5 expression or miR-455-5p overexpression improved neurobehavior and decreased apoptosis and oxidative injury (Wu et al., 2021).

#### LncRNA Small Nucleolar RNA Host Genes

SNHGs are a group of LncRNAs that are overexpressed in various cancers. They include SNHG1, SNHG3, SNHG5, SNHG6, SNHG7, SNHG12, SNHG15, SNHG16, and SNHG20 (Zimta et al., 2020). It has been reported that SNHG6 functions as a ceRNA for miR-181c-5p to regulate Bcl2 interacting mediator of cell death (BIM) expression and promote apoptosis (Zhang X. et al., 2019). In a previous study, SNHG7 expression was downregulated in C57 mice and PC12 cells. Additionally, SNHG7 overexpression or suppression of miR-9 expression resulted in decreased reactive oxygen species and malondialdehyde levels and increased cell viability. It has been reported that SNHG7 reduces OGD/R-induced neuronal apoptosis and functions as a ceRNA for miR-9 and SIRT1 targeted by miR-9. In summary, LncRNA SNHG7 serves as a ceRNA for miR-9 to regulate SIRT1 activity, thus alleviating neuronal injury (Zhou et al., 2020).

In another study, SNHG12 was highly expressed in SH-SY5Y cells subjected to OGD/R. Furthermore, SNHG12 targeted miR-181a-5p and negatively regulated its expression. Moreover, miR-181a-5p is a target of nerve growth factor receptor 1 (NEGR1) and negatively regulates the expression of NEGR1 in OGD-induced neuronal apoptosis (Yan Y. et al., 2020). SNHG15 expression was also upregulated in C57 mice, neuro-2a (N2a) cells, and PC12 cells in previous studies (Guo et al., 2020; Fan et al., 2021). Guo et al. found that silencing SNHG15 resulted in upregulation of the expression of C-X-C motif chemokine ligand 13 (CXCL13) through suppression of the activation of miR-18a and extracellular signal-regulated kinase (ERK)/mitogen-activated protein kinase kinase (MEK). These resulted in reduced apoptosis and enhanced cell viability (Guo et al., 2020). Furthermore, Fan et al. revealed that downregulation of LncRNA SHNG15 expression plays a protective role in OGD/R-induced neuronal injury through downregulation of the expression of tumor protein p53 inducible nuclear protein 1 (TP53INP1) *via* miR-445-3p targeting (Fan et al., 2021).

#### LncRNA Rhabdomyosarcoma 2-Associated Transcript

The expression of LncRNA RMST was upregulated whereas that of miR-377 was downregulated in serum samples collected from patients with IS. Furthermore, suppression of RMST expression resulted in reduced oxidative stress and apoptosis in an N2a cell culture model of OGD. Semaphorin 3A (SEMA3A) is a target gene of miR-377, and RMST regulates SEMA3A expression as a sponge for miR-377 (Zhao et al., 2021). A previous study revealed that RMST interacts with heterogeneous nuclear ribonucleoprotein K (hnRNPK) and then regulates the p53/miR-107 axis, thus promoting apoptosis of HT-22 cells (Cheng et al., 2020).

#### LncRNA Antisense Non-Coding RNA in the INK4 Locus

It has been reported that LncRNA ANRIL is involved in neuronal apoptosis, and its expression was decreased in patients with acute IS, rat, and mouse models of MCAO/R, (Feng et al., 2019; Liu et al., 2019; Zhong et al., 2020). Interestingly, one study demonstrated that the level of LncRNA ANRIL in N2a cells significantly decreased within 12 h after OGD but increased at 18 h and peaked at 24 h after OGD (Zhong et al., 2020). Another study also revealed that LncRNA ANRIL expression is downregulated within 8 h after OGD in PC12 cells (Liu et al., 2019). Furthermore, silencing ANRIL aggravated OGD-induced PC12 cell injury *via* the reduction in cell viability and increase in apoptosis, while overexpression of ANRIL contributed to opposite effects. Overexpression of miR-127 and myeloid cell leukemia-1 (Mcl-1) results in significantly enhanced cell injury. Additionally, miR-127 negatively regulates Mcl-1 expression, whereas ANRIL upregulates Mcl-1 expression by downregulating miR-127 expression (Liu et al., 2019). It has been revealed that ANRIL competitively interacts with miR-199a-5p in N2a cells. Additionally, overexpression of ANRIL or suppression of miR-199a-5p expression results in the protection of cells against I/R injury and improved cell viability through the caveolin-1 (CAV-1)-mediated MEK/ERK pathway (Zhong et al., 2020).

#### LncRNA MALAT1

LncRNA MALAT1 has been reported to be highly expressed in both *in vivo* and *in vitro* models of ischemia (Guo et al., 2017; Zhang G. et al., 2020; Jia et al., 2021). In addition, It has also been claimed that downregulation of MALAT1 expression alleviates neuronal apoptosis. Furthermore, suppression of MALAT1 expression or overexpression of miR-375 leads to a decrease in the levels of inflammatory factors and lactate dehydrogenase as well as a reduction in apoptosis. Furthermore, miR-375 targets both MALAT1 and phosphodiesterase 4D (PDE4D) (Zhang G. et al., 2020). It has also been reported that MALAT1 knockdown causes a reversal in OGD/R-induced apoptosis and endoplasmic reticulum stress through the targeting of miR-195a-5p and regulating the expression of high mobility group AT-hook1 (HMGA1) (Jia et al., 2021).

#### LncRNA Potassium Voltage-Gated Channel Subfamily Q Member one Opposite Strand 1 (KCNQ1OT1)

KCNQ1OT1 was highly expressed in patients with acute IS, MCAO/R models, and OGD cell culture models in a previous study. Further, downregulation of KCNQ1OT1 expression significantly ameliorated apoptosis in OGD/R treated PC12 cells. It has also been reported that KCNQ1OT1 serves as a ceRNA for miR-140-3p and regulates the expression of hypoxia-inducible factor (HIF)-1α, which is a target of miR-140-3p (Yi et al., 2020). KCNQ1OT1 and MMP8 expressions were significantly increased in neurons but miR-9 was downregulated. Inhibiting KCNQ1OT1 or overexpressing miR-9 protected OGD/R-induced neuronal injury (Wang HJ. et al., 2020; Ren et al., 2020). Another study revealed KCNQ1OT1 promoted neuronal apoptosis *via* miR-153-3p/forkhead box O3 (FOXO3) axis (Wang HJ. et al., 2020).

#### Other LncRNAs

Other LncRNA-mediated ceRNA networks have been studied in neuronal cell cultures. For instance, LncRNA taurine-upregulated gene 1 (TUG1) has been shown to play an important role in apoptosis in IS. TUG1 expression was found to be upregulated in cultured neurons under OGD insult. Additionally, knockdown of TUG1 reportedly results in decreased apoptosis and increased cell survival *in vitro*. It has been found that TUG1 directly binds to miR-9 and that downregulating miR-9 expression reverses the suppressive effect of TUG1 on B-cell lymphoma-2 like-11 (Bcl2l11) expression (Chen et al., 2017). In a study conducted by Wang et al., LncRNA HOXA transcript at the distal tip (HOTTIP) was found to induce neuronal injury by modulating the miR-143/hexokinase 2 pathway (Wang Y. et al., 2018). In other studies, the LncRNA Gm11974/miR-766-3p/nuclear receptor subfamily 3 group C member 2 (NR3C2) and LncRNA cardiac hypertrophy-related factor (CHRF)/miR-126/sex-determining region Y box 6 (SOX6) axes protected against cerebral I/R injury and reduce neuronal apoptosis (Cai et al., 2019; Gai et al., 2019). Jing et al. also found that overexpression of LncRNA mu-1-opioid peptide receptor (Oprm1) results in the alleviation of apoptosis from cerebral I/R injury through the Oprm1/miR-155/GATA binding protein 3 (GATA3) axis (Jing et al., 2019). Furthermore, the LncRNA AK038897/miR-26a-5p/death-associated protein kinase 1 (DAPK1), FGD5 antisense RNA 1 (FGD5-AS1)/miRNA-223/insulin-like growth factor (IGF)-1 receptor (IGF1R), tumor-associated LncRNA expressed on chromosome 2 (TALNEC2)/miR-650/apoptotic peptidase activating factor 1 (APAF1), FOXD3-AS1/miR-765/BCL2L13, and RNA imprinted and accumulated in nucleus (Rian)/miR-144-3p/GATA3 axes have been found to attenuate apoptosis after cerebral I/R injury (Zhang XQ. et al., 2019; Wei et al., 2019; Lu et al., 2020; Yao et al., 2020; Chen M. et al., 2021; Cao et al., 2021).

### Autophagy

Several ceRNA networks were related to the autophagy of neurons. Luo et al. suggested that the MEG3/miR-378/growth factor receptor-bound protein 2 (GRB2) axis plays a role in neuronal autophagy and impairment of neurological function in IS (Luo et al., 2020). Knockdown of KCNQ1OT1 resulted in reduced infarct volume in mice subjected to MCAO as well as neuronal autophagy *via* the miR-200a/forkhead box O3 (FOXO3)/autophagy-related protein 7 (ATG7) axis (Yu et al., 2019). Downregulation of MALAT1 expression was shown to result in ischemic injury and autophagy suppression. MALAT1 served as a molecular sponge for miR-30a and interfered with the inhibitory effect of miR-30a on ischemic injury and autophagy by suppressing the expression of Beclin-1, which is a direct target of miR-30a. Altogether, suppression of MALAT1 expression attenuates neuronal cell death *via* the miR-30a/Beclin-1 axis (Guo et al., 2017). LncRNA SNHG14 and BINP3, a pro-apoptotic mitochondrial protein, were upregulated in OGD/R-induced HT22 cells, but miR-182-5p was downregulated. What’s more, SNHG14 could regulate the expression of BNIP3 *via* targeting to miR-182-5p. Overall, SNHG14 promoted mitophagy *via* miR-182-5p/BINP3(Deng et al., 2020).

### Inflammation

In a previous study, LncRNA SNHG14 was found upregulated in MCAO/R rats and OGD/R-induced PC-12 cells. SNHG14 acted as a sponge of miR-136-5p and positively regulated the expression of Rho-associated coiled-coil-containing protein kinase 1 (ROCK1), thus promoting neurological impairment and inflammation (Zhong et al., 2019). P65 is a subunit of nuclear factor NF-κB, which is related to an inflammatory response. Li et al. underlined that LncRNA H19 targeted p65 by sponging of miR-138-5p, thus promoting inflammatory response and improving neurological functions (Li et al., 2020).

## Regulatory Roles of LncRNAs in BMECs

**FIGURE 4:**
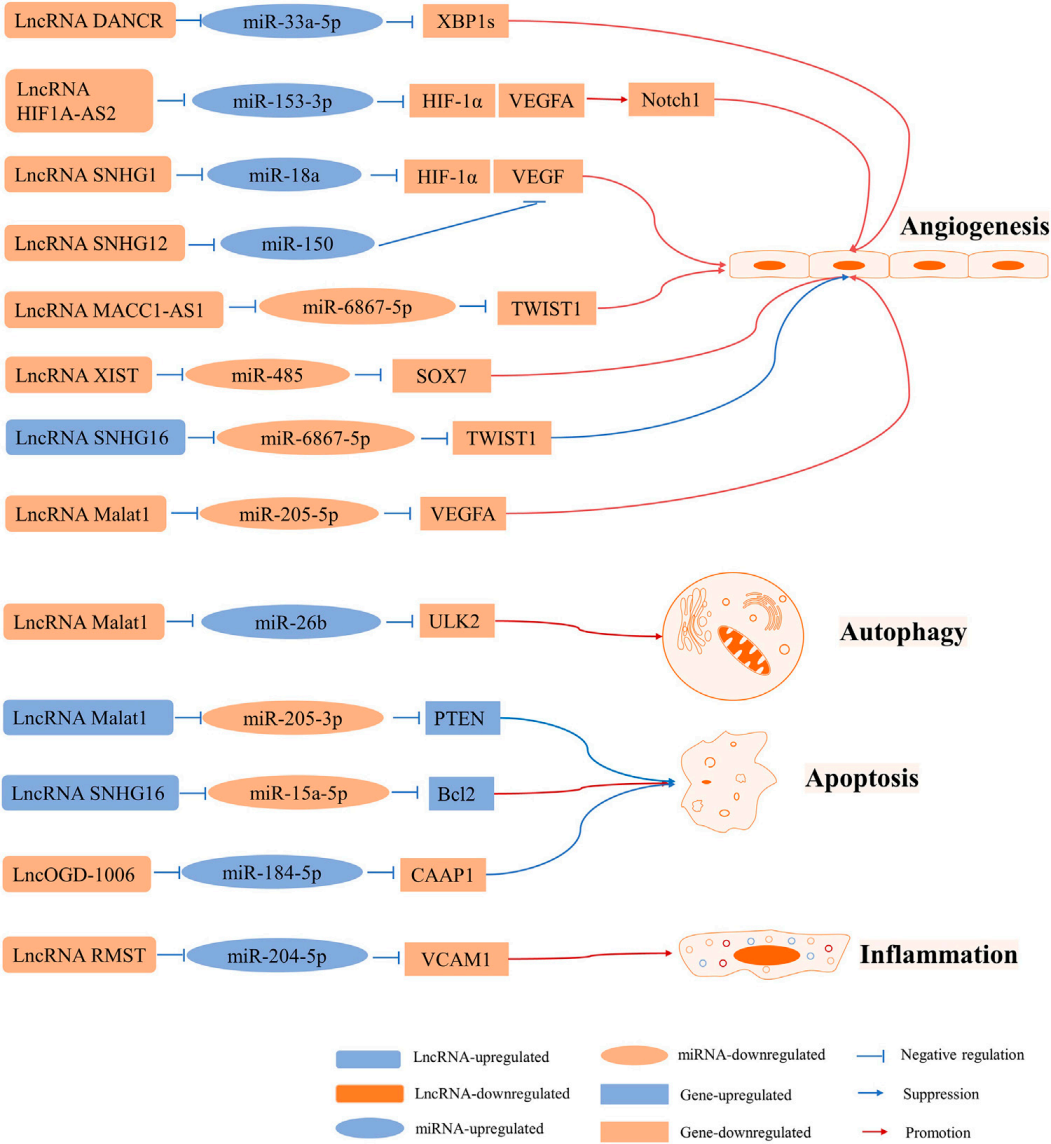
LncRNA-mediated ceRNA networks in BMECs involved in angiogenesis, autophagy, apoptosis, and inflammation.

BMECs are important components of the cerebral microvascular system and form a part of the blood-brain barrier (BBB). I/R injury leads to autophagy and apoptosis of BMECs, which accounts for BBB disruption and enhances vascular permeability, thus resulting in an unfavorable prognosis among patients suffering from IS (Li et al., 2014). Furthermore, vascular remodeling plays an important role in ischemic cardiovascular diseases. Angiogenesis in ischemic areas promotes blood supply to ischemic areas (Xu et al., 2018; Zhao et al., 2020). Thus, protecting BMECs from I/R injury or promoting angiogenesis can improve the prognosis of stroke (Zhu et al., 2021). In this section, we have discussed the roles of LncRNAs in BMECs after cerebral I/R injury (Figure 4).

### Autophagy and Apoptosis

LncRNA MALAT1 is one of the most highly upregulated I/R- or OGD/R-responsive endothelial LncRNAs that plays roles in apoptosis and inflammation (Zhang et al., 2017b; Yang et al., 2018; Zhang et al., 2018). One study revealed that MALAT1 promoted BMEC autophagy and survival by binding to miR-26b and downregulating its expression. Furthermore, miR-26b inhibited autophagy and cell survival, whereas overexpression of MALAT1 resulted in a reversal of this effect by promoting the expression of Unc-51 like autophagy activating kinase 2 (ULK2), a target of miR-26b (Li Z. et al., 2017). However, it was found that MALAT1 expression was downregulated in an OGD BMEC model. It was also claimed that MALAT1 can suppress apoptosis and function as a molecular sponge of miR-205-3p to modulate PTEN expression (Gao and Wang 2020). Altogether, MALAT1 can be a protective factor against BMEC injury. Furthermore, it has been reported that overexpression of miR-15a-5p results in decreased cell proliferation and increased apoptosis *via* downregulation of Bcl2 expression. It has been indicated that SNHG16 expression gradually decreases following OGD/R and that its overexpression results in the downregulation of miR-15a-5p expression, which promotes cell proliferation and decreases apoptosis. Overall, SNHG16 protects HBMECs from OGD/R-induced apoptosis *via* the miR-15a-5p/Bcl2 axis (Teng et al., 2020). In another study, LncRMST expression was upregulated in HBMECs and bEnd.3 cells subjected to OGD. Silencing LncOGD-1006 also aggravated OGD-induced injury in bEnd.3 cells *via* increased apoptosis, whereas overexpression of LncOGD-1006 led to opposite effects. Furthermore, LncOGD-1006 functions as a ceRNA for miR-184-5p to facilitate the expression of conserved anti-apoptotic protein 1 (CAAP1) (Chen JY. et al., 2020).

### Angiogenesis

Vascular endothelial growth factor (VEGF) is an angiogenesis inducer that promotes the growth of vascular endothelial cells. It has been reported that HIF-1 plays a key role in hypoxic responses and regulates VEGF expression (Ferrara 2004).

The LncRNA HIF1A-AS2 regulates the expression of HIF-1α by sponging miR-153-3p. In permanent MCAO and human umbilical vein endothelial cell (HUVEC) models, LncRNA HIF1A-AS2 expression is upregulated, whereas miR-153-3p expression is reduced as a result of higher protein levels of HIF-1α, VEGFA, and Notch1. Moreover, HUVEC viability, migration ability, and tube formation are promoted. Therefore, activating the LncRNA HIF1A-AS2/miR-153-3p/HIF-1α/VEGFA/Notch1 axis promotes angiogenesis in HUVECs (Li L. et al., 2017). Furthermore, SHNG12 upregulation or knockdown contributes to the regulation of VEGFA and fibroblast growth factor-beta mRNA and protein levels under OGD/R conditions. Additionally, capillary-like tube formation changes with SNHG12 expression, which indicates that SNHG12 promotes BMEC angiogenesis by targeting miR-199a (Long et al., 2018). It was shown in another study that SNHG12 improves angiogenesis following IS by regulating the miR-150/VEGF axis (Zhao et al., 2018). LncRNA Snhg1 promoted BMEC migration and tube formation after OGD insult in a previous study. Moreover, mechanistic studies have suggested that Snhg1 targets miR-338a and regulates HIF-1α and VEGF expression (Wang Z. et al., 2018). It has been reported that LncRNA MALAT1 protected against angiogenesis in HBMECs under OGD conditions via the miR-205-5p/VEGFA axis (Gao C. et al., 2020). Additionally, LncRNA metastasis-associated colon cancer 1 (MACC1)-AS1, which sponges miR-6867-5p/TWIST1, regulates the proliferation, survival, and migration of hypoxic HBMECs(Yan G. et al., 2020). Differentiation antagonizing non-protein coding RNA (DANCR) overexpression results in the promotion of spliced X-box binding protein l (XBP1s) expression in BMECs subjected to OGD. It has been shown that miR-33a-5p binds to DANCR and the 3ʹ-UTR of XBP1. Moreover, miR-33a-5p overexpression causes inhibition of cell proliferation, migration, and angiogenesis. In summary, DANCR promotes cell survival and angiogenesis by regulating the miR-33a-5p/XBP1s axis (Zhang M. et al., 2020).

### Inflammation

Vascular cell adhesion molecule 1 (VCAM1), an endothelial-specific marker, is related to inflammation in cerebrovascular disease (Maglinger et al., 2021; Wang et al., 2021). It has been shown that miR-204-5p is a target of VCAM1 and that RMST serves as a ceRNA that regulates VCAM1 expression by sponging miR-204-5p. Consequently, knockdown of RMST results in reduced OGD-induced injury through regulation of the miR-204-5p/VCAM1 axis (Yin et al., 2021).

## Regulatory Roles of LncRNAs in Microglia Activation and Polarization

**FIGURE 5:**
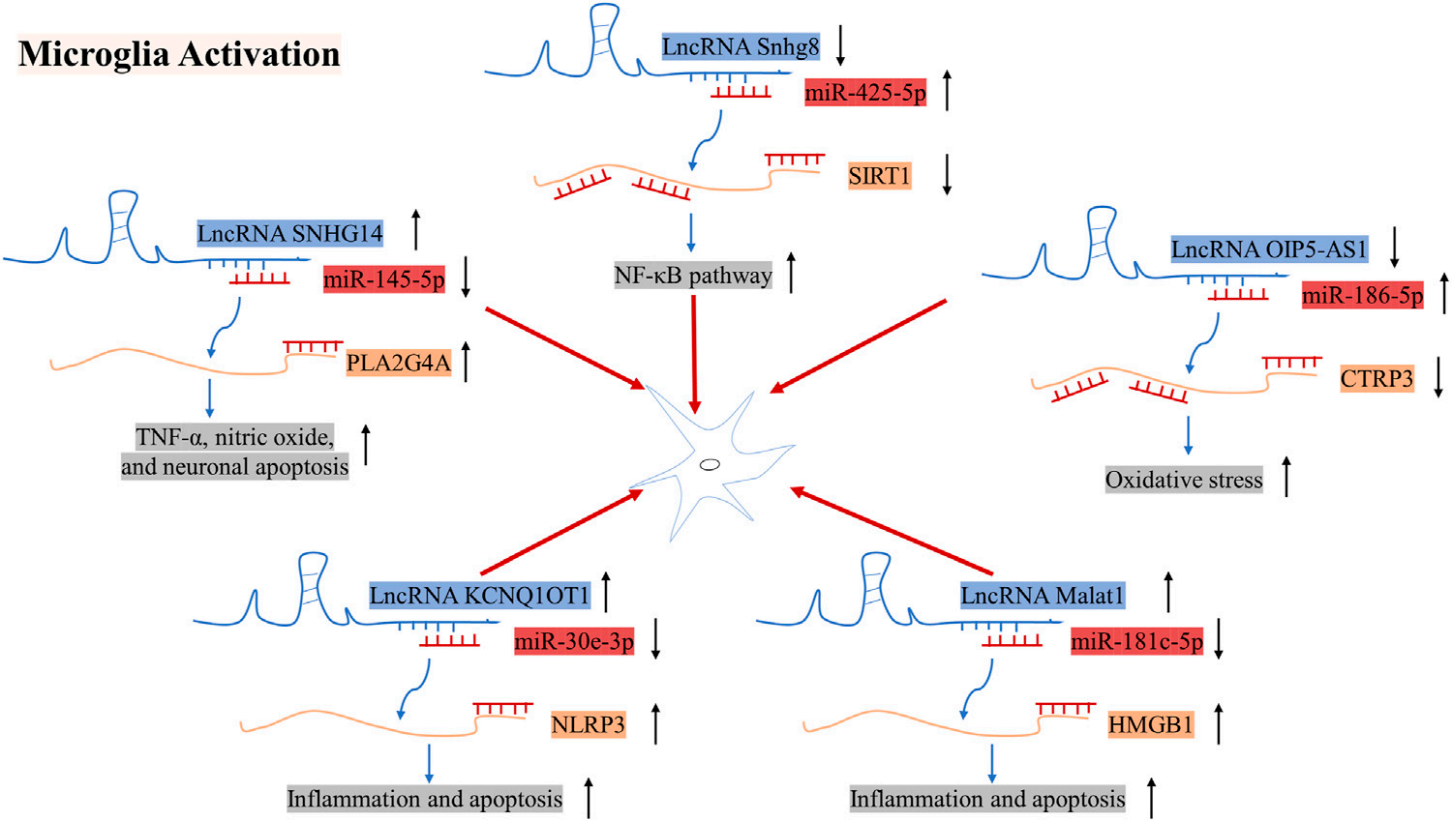
Mechanisms of action of LncRNA SNHG14, Snhg8, and OIP5-AS1 in microglial cells in the pathogenesis of IS. ↑ indicates upregulation, whereas ↓ indicates downregulation.

Resting microglia can be activated and polarized into two phenotypes in IS. M1 microglia produce pro-inflammatory mediators such as tumor necrosis factor α (TNF-α), interleukin (IL)-6, interferon-γ, IL-1β, inducible nitric oxide synthase, and proteolytic enzymes (matrix metalloproteinase [MMP] 9 and MMP3), which are harmful to brain tissues. Conversely, M2 microglia, which are also called tissue restorative microglia, produce pro-angiogenic and anti-inflammatory factors such as IL-10, transforming growth factor β, IGF, and VEGF, which promote ischemia (Qin et al., 2019). Here we have discussed the roles of several LncRNAs in microglial cells (Figure 5).

The expression of LncRNA Snhg8 was downregulated in ischemic regions in mice subjected to MCAO/R and in primary microglial cells subjected to OGD. Additionally, LncRNA Snhg8 serves as a ceRNA for miR-425-5p, which promotes microglial inflammation and BMEC injury by targeting the SIRT1/nuclear factor-κB (NF-κB) axis (Tian et al., 2021). The expression of the LncRNA SNHG14 was found to be strongly upregulated in mice with ischemic cerebral infarction and BV2 cells following OGD treatment. Gain and loss of function experiments revealed that SNHG14 regulates BV2 cell activation, TNF-α and nitric oxide production, and miR-145-5p and PLA2 group IVA (PLA2G4A) expression levels. Furthermore, overexpression of miR-145-5p caused a reversal of BV2 cell activation. Further studies have supported that SNHG14 directly binds to miR-145-5p and that the binding site of miR-145-5p exists on the 3ʹ-UTR of PLA2G4A. Overall, LncRNA SNHG14 promotes microglial activation by regulating the miR-145-5p/PLA2G4A axis (Qi et al., 2017). It has also been reported that the expression of LncRNA Opa-interacting protein 5 antisense RNA 1 (OIP5-AS1) and C1q/TNF-related protein 3 (CTRP3) is downregulated, while that of miR-186-5p is upregulated in BV2 cells subjected to OGD/R. Furthermore, it has been indicated that miR-186-5p promotes inflammation and oxidative stress in microglia and shares targets with CTRP3 and OIP5-AS1. Moreover, overexpression of LncRNA OIP5-AS1 promotes protection against I/R injury−induced inflammation and oxidative stress in microglia/macrophages *via* the miR-186-5p/CTRP3 axis (Chen Y. et al., 2021). Song. et al. found that the expression of LncRNA KCNQ1OT1 and NLRP3 was upregulated in HMC3 cells, while the expression of miR-30e-3p was downregulated. Further studies revealed that KCNQ1OT1 regulated cell inflammation and apoptosis by miR-30e-3p/NLRP3 pathway (Song et al., 2021). High-mobility group box 1(HMGB1) is a danger-associated molecular and takes part in inflammatory processes, which can be released under hypoxic and ischemic conditions. In previous studies, the expression of LncRNA Malat1 was augmented in MCAO/R mice. Further, Malat1 blocked the combination between miR-181c-5p and HMGB1, thus leading to an increase of the HMGB1 level. Totally, LncRNA Malat1/miR-181c-5p/HMGB1 axis may play a vital role in poststroke inflammation (Cao et al., 2020).

## Regulatory Roles of LncRNAs in Astrocytes

There are fewer studies on LncRNAs in astrocytes than in other cell lines. Aquaporin 4 (AQP4) is highly expressed in astrocytes and involved in the development of brain edema following intracerebral hemorrhage (Fu et al., 2007). It has been revealed that LncRNA MALAT1 exacerbates cerebral I/R injury, which regulates the expression of AQP4 by competitively binding to miR-375 (Wang H. et al., 2020). Moreover, LncRNA TUG1 aggravated apoptosis by upregulating AQP4 *via* miR-145 (Shan et al., 2020).

## LncRNAs in the Neurovascular Unit

Neuroprotective treatment strategies for IS face significant challenges in clinical settings. Numerous preclinical studies have demonstrated the potential benefits of neuroprotective therapy in animal models of IS. However, the clinical application of neuroprotective therapies tested in preclinical studies has mostly failed. Thus, it is critical to identify multi-target drugs to treat IS. The NVU is a multicellular complex composed of neurons, endothelial cells, astrocytes, myocytes, pericytes, microglia, and extracellular matrix. It is an intricate network that maintains a balanced neuronal microenvironment in the body (Muoio et al., 2014). LncRNAs that are widely expressed in cells of the NVU exert specific biological functions, which include multi-target and multi-link comprehensive regulation and precise regulation of the downstream network. However, to date, no study has investigated the relationship between LncRNAs and the NVU. In this section, we have summarized the roles of several LncRNAs expressed in different cells and identified their functions in IS.

### LncRNA MALAT1

The functions of LncRNA MALAT1 have been examined in different brain cells in previous studies. MALAT1 expression is upregulated after OGD/R treatment in neurons, BV-2 cells, and astrocytes, which results in MALAT1 acting as a harmful factor in I/R injury by promoting cell death *via* apoptosis and inflammation (Guo et al., 2017; Wang H. et al., 2020; Zhang G. et al., 2020; Cao et al., 2020; Jia et al., 2021). However, MALAT1 plays a protective role in BMECs subjected to OGD/R because it reduces apoptosis and promotes angiogenesis (Li Z. et al., 2017; Gao C. et al., 2020; Gao and Wang 2020). In summary, the roles of LncRNA MALAT1 in IS include a protective effect on endothelial cells; however, it may induce cell death in neurons and astrocytes.

### LncRNA RMST

It has been reported that the expression of LncRNA RMST is upregulated in HT22, bEnd.3, N2a, and BV2 cells, as well as in HBMECs and IS patients (Hou and Cheng 2018; Sun et al., 2019; Cheng et al., 2020; Yin et al., 2021; Zhao et al., 2021). Additionally, suppressing RMST expression results in reduced apoptosis of bEnd.3, HBMECs, and N2a cells, whereas overexpression of RMST promotes apoptosis of HT22 and BV2 cells. Collectively, these data indicate that LncRNA RMST may serve as an apoptosis promoter to regulate cerebral I/R injury.

## LncRNAs as Potential Diagnostic and Prognostic Biomarkers

**FIGURE 6:**
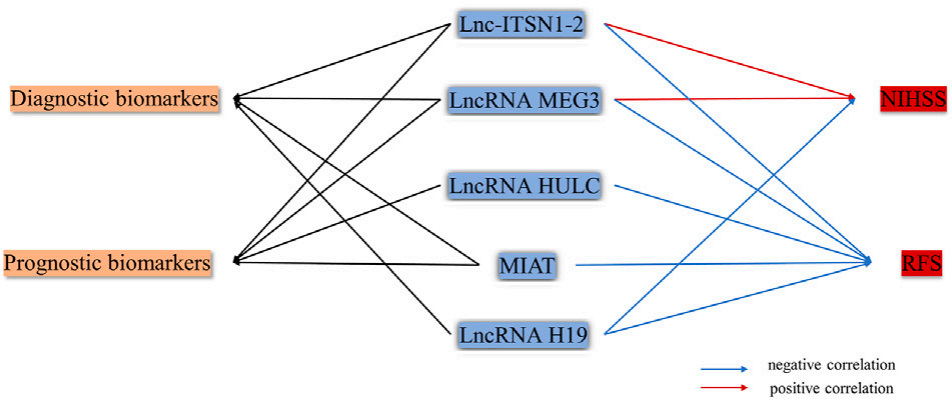
LncRNAs as potential diagnostic and prognostic biomarkers.

Some studies related to lncRNAs have been conducted in patients, in order to explore the clinical values (Figure 6). One study showed that lnc-ITSN1-2 expression was positively correlated with the National Institutes of Health Stroke Scale (NIHSS) score and reflected the severity of stroke. (Zhang and Niu 2020). Another case study was conducted to investigate the relationship between LncRNA MEG3 and prognostic value. MEG3 was upregulated in IS patients, positively associated with the NIHSS score, and negatively related to the prognosis of IS patients (Wang M. et al., 2020). Thus, MEG3 is a potential marker for diagnosis and prognosis. For prognosis, high lnc-ITSN1-2 expression was correlated with worse Relapse-Free Survival (RFS) in AIS patients (Zhang and Niu 2020). Higher Myocardial infarction associated transcript (MIAT) expression had a relatively poor prognosis. Meanwhile, the multivariate analysis revealed that MIAT was an independent prognostic marker of functional outcome and death in patients with IS. Data suggested that MIAT might be a potential diagnostic and prognostic indicator in IS (Zhu et al., 2018). LncRNA HULC is correlated with higher AIS risk, increased disease severity, and worse prognosis in AIS patients (Ren et al., 2021). Meanwhile, it associates with higher IL-6, elevated intercellular adhesion molecule 1 (ICAM1), and lower miR-9 AIS patients (Chen X. et al., 2020). The H19 gene plays a functional role in increasing the prevalence of IS risk factors. The upregulation of H19 may be considered as a diagnostic biomarker in IS among the Iranian population. But ROC curve analysis revealed that the peripheral blood expression level of H19 could not be considered as a promising marker for the functional outcome and mortality prediction of IS patients, thus it cannot serve as a useful prognostic marker (Rezaei et al., 2021).

## Advantages and Limitations of Using LncRNAs as Therapeutics for IS

Nucleic acid-based RNA targeting approaches have been concerned by researchers. CeRNA networks provide a variety of therapies, such as ncRNA replacement therapy and ncRNA inhibition therapy (Dragomir et al., 2020). NcRNA replacement therapy is devoted to improving conditions of IS patients by supplementing ncRNAs. NcRNA inhibition therapy focuses on inhibiting the expression of harmful ncRNAs to slow the progress of IS. Furthermore, LncRNAs can be detected in the serum, which is more efficient in supporting the diagnosis and classification of IS patients.

However, the clinical application of LncRNAs has several limitations. Firstly, besides ceRNA network mechanisms, lncRNAs regulate gene expressions at multiple levels, including regulating chromatin modification, post-transcription, and transcription. Secondly, the levels of LncRNA, miRNA, and mRNA can be influenced by other molecular counterpart conditions, which makes it difficult to fully explore the intrinsic regulation mechanism of ceRNA and therefore require further investigations. Thirdly, since lncRNAs are not highly conserved among species, studies are mostly conducted in animal and cell models. Thus, there is a lack of reports on clinical studies on LncRNAs. Forthly, The development of ncRNA drugs is facing great difficulties, such as the degradation and delivery of ncRNA drugs. Finally, there is no sufficient information about exosomal lncRNAs in stroke pathogenesis, thus the functions of LncRNAs in NUV remain unclear and considerable research is needed in this field.

## Conclusion

The pathological process of cerebral I/R injury is complex. The lack of oxygen and energy causes ion pump failure, apoptosis, inflammation, glutamate excitatory toxicity, and oxidative stress, which are harmful to the cell components of NVU, such as neurons, glial cells, BMECs, and astrocytes. Previous studies have identified LncRNAs that are related to the occurrence and progression of IS. In this review, we first summarized how the LncRNA-mediated ceRNA networks take part in the process of ischemic stroke in different brain cell lines. Available data indicate that LncRNA MALAT1, MEG3, and RMST serving as ceRNAs, play an important role in neuroprotection, which mainly involves regulation of neuronal cell death by targeting miRNAs and mRNAs, such as PDC4D, caspases, SIRT1, and Beclin-1. Furthermore, some studies have identified the anti-apoptotic, anti-inflammatory, and angiogenic roles of LncRNAs in BMECs. Several LncRNAs function as ceRNAs to bind to miRNAs and regulate the expression of VEGF, which is an important angiogenic factor. Some ceRNA networks are also involved in the activation and polarization of microglia, whereas others can regulate the expression of inflammatory factors. Secondly, we analyzed the functions of LncRNAs that are expressed in different types of brain cells to better understand the relationship between LncRNAs and the NVU. Thirdly, some researchers have shown that the expression level of LncRNAs is related to neurological deficits and prognosis among IS patients. These results indicate that LncRNAs may be potential therapeutic targets and biomarkers. What’s more, lncRNA mediated ceRNA networks are involved in different pathological mechanisms in aggravating I/R damage; however, the underlying mechanisms are not fully understood. Although studies related to lncRNAs have been conducted based on clinical samples, the differences between different races and nationalities blocked lncRNAs from being effective IS markers in diagnosing and prognosing. More studies should be conducted to explore the clinical and medical value of lncRNAs or ceRNAs in IS.

## Glossary

3ʹ-UTR: 3ʹ-untranslated region
AIM2: absent in melanoma 2
ANRIL: antisense non-coding RNA in the INK4 locus
APAF1: apoptotic peptidase activating factor 1
AQP4: aquaporin 4
ATG7: autophagy related protein 7
BBB: blood-brain-barrier
Bcl2: B-cell lymphoma-2
BMEC: brain microvascular endothelial cell
BIM: Bcl2 interacting mediator of cell death
CAAP1: conserved anti-apoptotic protein 1
CAV-1: caveolin-1
ceRNA: competing endogenous RNA
CHRF: cardiac hypertrophy-related factor
CTRP3: C1q/TNF-related protein 3
CXCL13: C-X-C motif chemokine ligand 13
DANCR: differentiation antagonizing non-protein coding RNA
DAPK1: death-associated protein kinase 1
ERK: extracellular signal-regulated kinase
FGD5-AS1: FGD5 antisense RNA 1
FOXD3-AS1: FOXD3 antisense RNA 1
FOXO3: forkhead box O3
GAS5: growth arrest-specific 5
GATA3: GATA binding protein 3
GRB2,: growth factor receptor-bound protein 2
HIF-1: hypoxia-inducible factor 1
HMGA1: high mobility group AT-hook1
hnRNPK: heterogeneous nuclear ribonucleoprotein K
HOTTIP: HOXA transcript at the distal tip
HUVEC: human umbilical vein endothelial cell
I/R: ischemia-reperfusion
Id2: inhibitor of DNA binding 2
IGF: insulin-like growth factor
IGF1R: IGF-1 receptor
IL: interleukin
IS: ischemic stroke
KCNQ1OT1: potassium voltage-gated channel subfamily Q member 1 opposite strand 1
LncRNA: long non-coding RNA
MACC1: metastasis-associated colon cancer 1
MALAT1: metastasis associated lung adenocarcinoma transcript 1
MAPK: mitogen-activated protein kinase
MCAO/R: middle cerebral artery occlusion/reperfusion
Mcl-1: myeloid cell leukemia-1
MEG3: maternally expressed gene 3
MEK: mitogen-activated protein kinase
miRNA: microRNA
MMP: matrix metalloproteinase
MRE: microRNA response element
mTOR: mechanistic target of rapamycin
N2a: neuro-2a
NEGR1: neuronal growth regulator 1
NO: nitric oxide
NF-κB: nuclear factor-κB
NR3C2: nuclear receptor subfamily 3 group C member 2
NVU: neurovascular unit
OGD/R: oxygen-glucose deprivation and reoxygenation
OIP5-AS1: Opa-interacting protein 5 antisense transcript 1
Oprm1: opioid receptor mu 1
PDCD4: programmed cell death 4
PDE4D: phosphodiesterase 4D
PGC-1α: peroxisome proliferator-activated receptor-gamma coactivator 1 alpha
PLA2G4A: phospholipase A2 group IVA
PTEN: phosphatase and tensin homolog deleted on chromosome ten
PUMA: p53 upregulated modulator of apoptosis
Rian: RNA imprinted and accumulated in nucleus
RMST: rhabdomyosarcoma 2-associated transcript
SD: Sprague Dawley
SEMA3A: semaphorin 3A
SIRT1: sirtuin1
SNHGs: small nucleolar RNA host genes
SOX6: sex-determining region Y box six
TALNEC2: tumor-associated lncRNA expressed on chromosome two
TNF-α: tumor necrosis factor α
TP53INP1: tumor protein p53 inducible nuclear protein one
TUG1: taurine upregulated gene one
ULK2: Unc-51 like autophagy activating kinase two
VCAM1: vascular cell adhesion molecule one
VEGF: vascular endothelial growth factor
XBP1s: X-box binding protein 1
